# Percutaneous balloon kyphoplasty for the treatment of vertebral compression fractures

**DOI:** 10.1186/1471-2482-14-3

**Published:** 2014-01-14

**Authors:** Chia-Wei Yu, Ming-Kai Hsieh, Lih-Huei Chen, Chi-Chien Niu, Tsai-Sheng Fu, Po-Liang Lai, Wen-Jer Chen, Wen-Chien Chen, Meng-Ling Lu

**Affiliations:** 1 Department of Orthopaedic Surgery, Chang Gung Memorial Hospital & Chang Gung University, 5, Fu-Hsin Street, Kweishan Shiang, Taoyuan 333, Taiwan

## Abstract

**Background:**

Vertebral compression fractures (VCFs) constitute a major health care problem, not only because of their high incidence but also because of their direct and indirect negative impacts on both patients’ health-related quality of life and costs to the health care system. Two minimally invasive surgical approaches were developed for the management of symptomatic VCFs: balloon kyphoplasty and vertebroplasty. The purpose of this study was to evaluate the effectiveness and safety of balloon kyphoplasty in the treatment of symptomatic VCFs.

**Methods:**

Between July 2011 and June 2012, one hundred and eighty-seven patients with two hundred and fifty-one vertebras received balloon kyphoplasty in our hospital. There were sixty-five male and one hundred and twenty-two female patients with an average age of 74.5 (range, 61 to 95 years). The pain symptoms and quality of life, were measured before operation and at one day, three months, six months and one year following kyphoplasty. Radiographic data including restoration of kyphotic angle, anterior vertebral height, and any leakage of cement were defined.

**Results:**

The mean visual analog pain scale decreased from a preoperative value of 7.7 to 2.2 at one day (p < .05) following operation and the Oswestry Disability Index improved from 56.8 to 18.3 (p < .05). The kyphotic angle improved from a mean of 14.4° before surgery to 6.7° at one day after surgery (p < .05). The mean anterior vertebral height increased significantly from 52% before surgery to 74.5% at one day after surgery (p < .05) and 70.2% at one year follow-up. Minor cement extravasations were observed in twenty-nine out of two hundred and fifty-one procedures, including six leakage via basivertebral vein, three leakage via segmental vein and twenty leakage through a cortical defect. None of the leakages were associated with any clinical consequences.

**Conclusions:**

Balloon kyphoplasty not only rapidly reduced pain and disability but also restored sagittal alignment in our patients at one-year follow-up. The treatment of osteoporotic vertebral compression fractures with balloon kyphoplasty is a safe, effective, and minimally invasive procedure that provides satisfactory clinical results.

## Background

Vertebral compression fractures (VCFs) constitute a major health care problem, not only because of their high incidence but also because of their direct and indirect negative impacts on both patients’ health-related quality of life and costs to the health care system [1,2]. Approximately 26% of women aged > fifty years and 40% of women aged > eighty years are reported to have sustained a VCF [3,4]. Furthermore, one-third to three-fourths of such patients subsequently develop chronic back pain, which can be attributed to pseudarthrosis or osteoporotic spinal deformity, such as kyphosis or kyphoscoliosis [1]. The degree of kyphosis correlates well with the patient’s physical function, risk of further fracture [5,6], compression of the spinal cord, mental well-being, and pulmonary function [7], any of which can contribute to an increased mortality rate [8,9].

Regardless of their etiology, the mainstay of management for symptomatic VCFs is medical therapy, which may include analgesics, bed rest, external fixation such as brace or corset, and/or rehabilitation [10,11]. However, anti-inflammatory drugs and certain types of analgesics are poorly tolerated by elderly patients, and bed rest can lead to further bone demineralization, which may predispose to future fractures as well as thromboembolic complications and pneumonia. Furthermore, surgical fixation often fails due to the poor quality of osteoporotic bone [12], and because of the risks of open surgery in this predominantly elderly population, these procedures are generally limited to cases in which there is concurrent spinal instability and/or neurologic deficit [12,13].

Because of these limitations, two minimally invasive surgical approaches were developed for the management of symptomatic VCFs: balloon kyphoplasty and vertebroplasty [14,15]. Vertebroplasty, which was first described by Galibert and colleagues [16] in 1987, involves the percutaneous injection of viscous polymethylmethacrylate (PMMA) into the vertebral body. In balloon kyphoplasty, injection of PMMA follows insertion of a tamp (balloon) into the vertebral body, using either a transpedicular or extrapedicular route, in order to compress the cancellous bone, create a cavity, and if possible, realign the endplate of the vertebral body. After removal of the bone tamp, the PMMA fixes and stabilizes the fracture. The purpose of this study was to evaluate the effectiveness and safety of balloon kyphoplasty in the treatment of symptomatic VCFs.

## Methods

### Patients

Between July 2011 and June 2012, one hundred and eighty-seven patients with two hundred and fifty-one vertebras received balloon kyphoplasty in our hospital. The Chang Gung Medical Foundation Institutional Review Board approved this study (101-3955B) and waived the requirement for informed consent due to the retrospective nature of the study. All patients met the following criteria: (1) focal midline back pain managed inadequately with appropriate conservative treatment, (2) back pain related to VCF location on radiography, and (3) presence of bone marrow edema on magnetic resonance imaging (MRI), indicated by hypointense signal on T1-weighted images and hyperintense signal on T2-weighted images. Exclusion criteria were active infection, neurologic deficit, and uncorrected therapeutic anticoagulation. About four hundred patients per year were treated with vertebroplasty or kyphoplasty in our department. Kyphoplasty was favored in the presence of kyphotic deformity which contribute significantly to morbidity and disruption of the posterior vertebral cortex, where high-viscosity cement can be delivered.

The study population comprised one hundred and eighty-seven patients: sixty-five men (35%) and one hundred and twenty-two women (65%), whose median age was 74.5 years (range, 61–95 years). Demographic data, including cause of fracture, fracture location, treated vertebra (e) per patient, and time from fracture to kyphoplasty, were recorded (Table 1). Clinical follow-up examination of the patients was independently performed by an orthopedic specialist, and diagnostic images were independently evaluated by a radiologist.

**Table 1:** Patient demographics and clinical data

**Characteristic**	**No. (%)**
Age (y) (n = 187 patients)	
60-69	35(18.7%)
70-79	74(39.5%)
80-89	66(35.3%)
90-99	12(6.5%)
Sex (n = 187 patients)	
Female	122(65%)
Male	65(35%)
No. of fractures treated (n = 187 patients)	
1	138(73.9%)
2	37(19.7%)
3	9(4.8%)
4	3(1.6%)
Cause of fracture (n = 187 patients)	
Osteoporosis	181(96.8%)
Metastasis	1(0.5%)
Multiple myeloma	5(2.7%)
Time from fracture to kyphoplasty (n = 187 patients)	
Acute (0–2 weeks)	18(9.6%)
Subacute (2 weeks–3 months)	93(49.7%)
Chronic (>3 months)	76(40.7%)
Time from kyphoplasty to latest OPD F/U (n = 187 patients)	
≧12 months	183(97.9%)
Loss of follow -up	4(2.1%)

### Surgical technique

**Figure 1:**
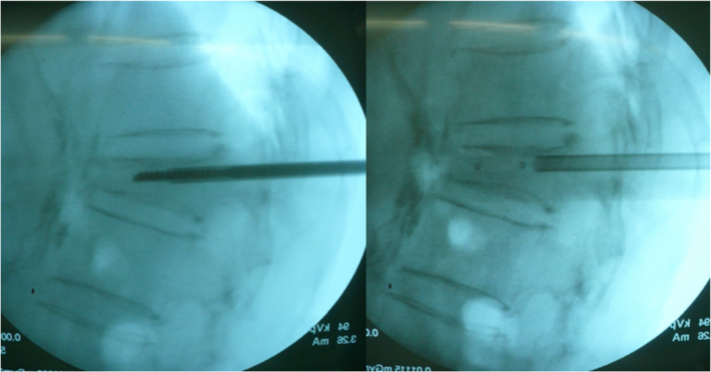
**After a Niddle Pipe was placed via a stab incision.** A Cannular and Expander was inserted into the pedicle through the Wire-Pin and slowly inflate the balloon with initial bulk-pressure.

**Figure 2:**
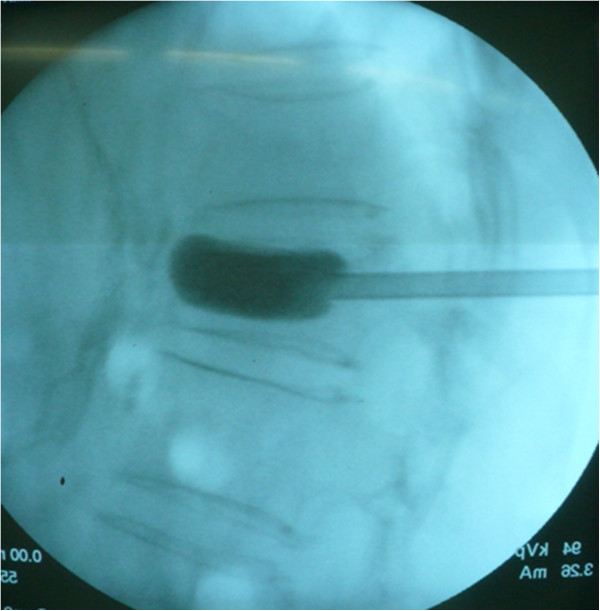
**The operator controls the volume of the Ballon to recover the damaged vertebral body with micro-pressure until adequate kyphotic angle reduction is obtained or the inflation pressure reached 220 psi.** The operator should record the amount of injected fluid to predict the cement volume.

**Figure 3:**
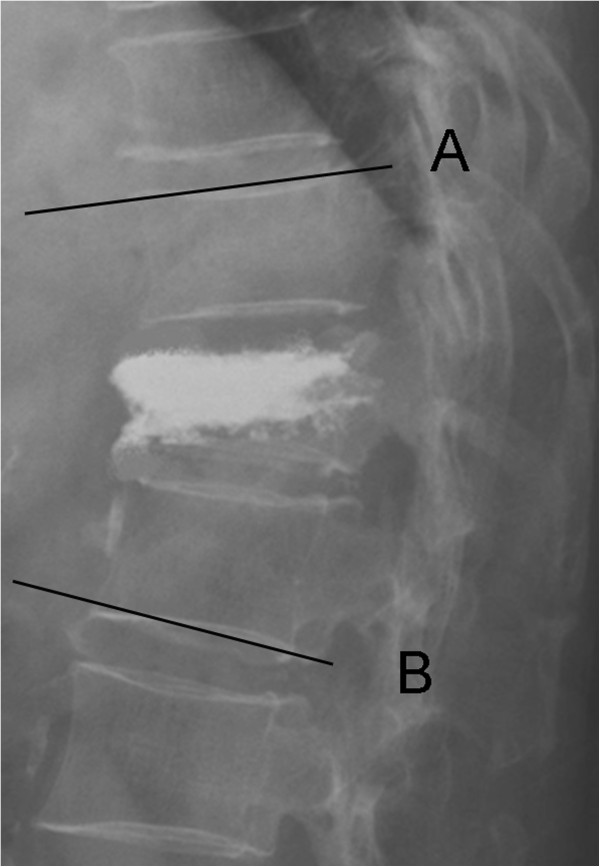
**The balloon is deflated and withdrawn, and the resulting intravertebral cavity is filled with PMMA cement.** Kyphotic angle defined as the Cobb angle measurements taken from the superior endplate of the vertebra one level above the treated vertebra (Line **A**) to the inferior endplate of the vertebral body one level below the treated vertebra (Line **B**) on the lateral X-ray image.

Percutaneous kyphoplasty was performed under local anesthesia with the patient positioned prone on a radiolucent table with his/her spine extended by chest and pelvic bolsters. A preoperative prophylactic single-shot intravenous dose of a first-generation cephalosporin was administered to each patient, and fluoroscopy was used throughout the procedure. A stab incision was made on the pedicle level of the skin; the correct incision site was identified using the anteroposterior (AP) view of the image intensifier. A needle pipe and pin were then placed with the tip lateral to the pedicle projection in the AP view and parallel to the superior endplate in the lateral view. Then, the needle pin was removed, and a wire pin was introduced into two-thirds of the vertebral body; subsequently, the needle pipe was removed. A cannula and expander were inserted into the pedicle through the wire pin (Figure 1). The wire pin was removed, and a drill was inserted through the cannula. An inflatable balloon (Veresys; SI MEDICAL CO., LTD., Korea) was inserted unilaterally into the fractured vertebral body and slowly inflated with initial bulk pressure (Figure 2). The operator controlled the volume of the balloon to restore the damaged vertebral body with micropressure until adequate kyphotic angle reduction was obtained or when the inflation pressure reached two hundred and twenty psi. The operator then recorded the amount of injected fluid to predict the cement volume (Figure 3). Thereafter, the inflated balloon was deflated and withdrawn (Figure), and the resultant intravertebral cavity was filled with PMMA cement (SimplexP; Stryker Howmedica Osteonics, Allendale, NJ, USA) (Figure).

### Operative and radiographic outcomes

Parameters related to imaging and technical characteristics, including the approach method, amount of bone cement injected, and local or general anesthesia applied were recorded (Table 2). Restoration of kyphotic angle which was defined as the Cobb angle measurements taken from the superior endplate of the vertebra one level above the treated vertebra to the inferior endplate of the vertebral body one level below the treated vertebra on the lateral X-ray image, height of the anterior border of the collapsed vertebral body, and any leakage of cement, were determined. Patterns of cement leakage were assessed by radiographs using the classification proposed by Yeom and colleagues [17], which identifies three leakage sites (Figure 2): (1) via the basivertebral vein (B type), (2) via the segmental vein (S type), or (3) through a cortical defect (C type).

**Table 2:** Operative characteristics

**Operative Characteristic**	**No. (%)**
Approach (n = 187 patients)	
Unilateral extrapedicular	184(98.4%)
Bipedicular	3(1.6%)
Volume injected (n = 251 vertebras)	
<3.5 cc	14(5.5%)
3.5-7 cc	203(81%)
>7 cc	34(13.5%)
Anesthesia (n = 187 patients)	
Local	178(95.2%)
General	9(4.8%)
Leakage (n = 251 vertebras)	
B type	6(2.4%)
S type	3(1.2%)
C type	20(7.9%)
Total	29(11.5%)

B type:leakage via basivertebral vein.

S type:leakage via segmental vein.

C type: leakage through a cortical defect.

### Clinical assessment

Clinical outcome was evaluated using the Oswestry Disability Index (ODI), and the visual analog scale (VAS) preoperatively and at the final follow-up. All patients were scheduled for follow-up at one day, three month, six month and one year after surgery, and annually thereafter.

### Statistical analysis

Data were analyzed using the SPSS statistical software package (IBM Corporation, Armonk, NY, USA). Means were calculated for different variables, including ODI, and VAS scores, kyphotic angle and height of the anterior border of the collapsed vertebral body. Preoperative and postoperative measurements and values between the different subgroups were compared using the paired *t* test, with statistical significance was set at P < .05.

## Results

### Operative and radiographic outcomes

Radiographic measurement of all the two hundred and fifty-one vertebrae treated demonstrated that the kyphotic angle improved from a mean of 14.4° ±2.2° before surgery to 6.7° ± 1.1° one day after surgery (p = .003) and to 7.6° ± 0.9° in two hundred and forty-seven vertebrae of one hundred and eighty-three patients at the last follow-up. The mean anterior vertebral height increased significantly from 52% ± 6.9% before surgery to74.5% ± 7.9% at one day after surgery (p = .0021) and 70.2% ± 5.2% at the last follow-up (Table 3).

**Table 3:** Radiographic and clinical data after kyphoplasty

	**Pre-op**	**Post-op day1**	**Post-op month 3**	**Post-op month 6**	**Post-op month 12**
Mean VAS score	7.7 ± 1.3	2.2 ± 0.9*	1.4 ± 0.6	0.8 ± 0.2	0.5 ± 0.1*
Mean ODI score	56.8 ± 4.2	18.3 ± 2.3*	17.3 ± 2.2	15.2 ± 1.9	12.5 ± 1.6*
AVH (%)	52 ± 6.9	74.5 ± 7.9*	72.4 ± 5.5	72.2 ± 4.5	70.2 ± 5.2
Kyphotic angle (°)	14.4 ± 2.2	6.7 ± 1.2*	7.2 ± 1.4	7.4 ± 1.1	7.6 ± 0.9

VAS: visual analogue scale;

ODI: Oswestry Disability Index;

AVH: anterior vertebral height, Anterior Vertebral height is expressed as fractions of referent vertebral height (AVH = Anterior Vertebral height of index fractured vertebrae/average of above and below intact anterior vertebral height),

*p < .05.

### Clinical outcome

One hundred and eighty-three patients finished one year follow-up till June 2013 and four patients were lost to follow-up in first 3 months. All of the One hundred and eighty-seven patients tolerated the procedure well. The mean operating time was 42.4 ± 15.5 minutes per vertebra. All of the patients experienced some degree of pain relief and improvement in mobility within the first twenty-four hours following surgery. The mean VAS score improved significantly from 7.7 ± 1.3 before surgery to 2.2 ± 0.9 at one day (p = .0012), and 0.5 ± 0.1 in one hundred and eighty-three patients at one year after surgery (p = .0021). The patients’ ODI score also improved significantly after surgery, which improved from 56.8 ± 4.2 to 18.3 ± 2.3 at one day (p = .0031) and 12.5 ± 1.6 at one year follow-up (p = .0012) (Table 3).

### Cement extravasation

The orthopedic balloon ruptured in two patients with no further consequences. The balloon was replaced, and the operation was continued in the usual manner. In all cases, a cavity was created successfully in the treated vertebral body. Minor cement extravasations were observed in twenty-nine out of two hundred and fifty-one procedures, including six leakages via basivertebral vein, three leakages via segmental vein and twenty leakages through a cortical defect (Table 2). None of the leakages were associated with any clinical consequences. None of the patients developed neurological deficits, symptomatic pulmonary embolism or postoperative infections.

## Discussion

Osteoporosis is a systemic disease that results from progressive bone mineral loss and changes in bony architecture, leaving the spinal column vulnerable to VCFs. Painful osteoporotic VCFs can be a significant burden for patients, as they impair physical function and quality of life. Moreover, VCFs can lead to progressive sagittal spine deformities and changes in spinal biomechanics, which are believed to contribute to a five-fold increased risk of further fracture [18].

Conservative treatment for the pain caused by VCFs includes analgesic medication, bed rest, and back braces; however, these therapies do not address spinal deformities. Furthermore, pain and disability may be prolonged while the fractured vertebral body heals [4]. In most cases, vertebral stabilization using open surgery is not indicated owing to increased risk for the patient and unsatisfactory results, such as loosened and lost screws in weak bone. In contrast, vertebroplasty is particularly advantageous because of its short surgical time, rapid pain relief, and minimal recovery period [19,20]. Moreover, vertebroplasty in osteoporotic VCFs, involving the percutaneous injection of cement directly into the fractured vertebra, is effective in ameliorating VCF-associated pain [21-23]. The limitations of this procedure include its inability to address the kyphotic deformity and the substantial risk of extravertebral cement leakage after the high-pressure cement injection. A balloon kyphoplasty, on the other hand, attempts to restore spinal alignment via a lower-pressure placement of cement into a cavity that is created in the vertebral body by a tamp inside the vertebral body. In addition to the available evidence, the benefits of balloon kyphoplasty based on our results included better spinal alignment restoration and less cement extravasation than those in vertebroplasty ndeed, previous studies have reported that kyphoplasty results in considerably less cement extravasation than does vertebroplasty [15,19,23-28]. In our series, the mean VAS score and ODI score improved significantly from 7.7 ± 1.3 to 0.5 ± 0.1 and from 56.8 ± 4.2 to 12.5 ± 1.6 at one year of follow-up, the long-lasting pain relief provided by balloon kyphoplasty can be attributed to the improved sagittal profile of the spine, which results in a lower compensating activity of the muscles [26,29].

The rapid relief of pain after balloon kyphoplasty can be associated with the effect of the bone cement and the stabilization of the vertebral body. Patients in this study exhibited a rapid decrease in pain, with a significant improvement in VAS score after surgery. The restoration of normal overall spinal sagittal alignment in elderly patients with VCFs and kyphotic deformities has obvious benefits [30]. In this series, an improvement in spinal sagittal alignment and vertebral body height was achieved in most patients. A mean correction of 7.7° was achieved in local spinal kyphosis; this is similar to the 8.8° reported in another series [28].

As previously reported, kyphoplasty results in considerably less cement extravasation than does vertebroplasty [15,24-27]. The rate of cement leakage was reported to be 9% following kyphoplasty and 41% following vertebroplasty in a systematic review of clinical studies [31]. An up-to-date meta-analysis reported cement extravasation in 7% of patients after kyphoplasty and in 20% after vertebroplasty [32]. In our study, asymptomatic cement extravasation occurred in an average of 11.5% of the vertebrae treated, which is considerably lower than that observed in vertebroplasty [9,23,25]. Compared with previous studies [9,23,25], this finding supports the fact that injection of high-viscosity cement at low pressure into a previously formed cavity is a significant improvement over the injection of low-viscosity cement at high pressure into an unreduced vertebral body. In our patients, cements with different viscosities were created as our experience increasing to treat different types of fractured vertebras.

The present study had limitations including its small number of patients and short follow-up period. The limited number of patients in this study possibly affects the statistical power and a long-term follow-up would be required to further evaluate the efficacy of this procedure. Our follow-up is only 12 months, which most authors consider to be a short-term period. However, the high rates of comorbidities and mortality related to elderly populations may make long-term follow-up infeasible.

We are currently gathering 2-year follow-up data from additional patients treated at our institution, but future prospective randomized studies with a large patient enrollment are needed to validate our findings.

## Conclusions

Kyphoplasty achieves good early results in the treatment of osteoporotic VCFs, with less bone cement leakage than that in vertebroplasty. Based on the results of our one-year follow-up, we conclude that balloon kyphoplasty rapidly reduced pain and disability and improved function and quality of life. Therefore, the treatment of VCFs with balloon kyphoplasty may be considered as is a safe, effective, and minimally invasive approach, as demonstrate by the satisfactory clinical results in our study.

## Competing interests

The authors declare that they have no competing interests.

## Authors’ contributions

Each author has made substantive intellectual contributions to this multicentre study: CWY and MKH participated in the study design, in collecting the data, the statistically analyses, draftin and contributed equally to the manuscript. CCN, TSF, PLL, WJC, WCC, and MLL, participated in the study design. LHC advised and assisted drafting of the manuscript. All authors read and approved the final manuscript.

## Pre-publication history

The pre-publication history for this paper can be accessed here:

http://www.biomedcentral.com/1471-2482/14/3/prepub
